# Social Networks in French Balneotherapy: A Focus on Spa Doctors

**DOI:** 10.3390/healthcare11192613

**Published:** 2023-09-22

**Authors:** Sybille Ramon Dupuy, Sandrine Cueille, Karine Dubourg, Christian-François Roques-Latrille, Frédéric Bauduer

**Affiliations:** 1 Institut du Thermalisme, University of Bordeaux, 40100 Dax, France; karine.dubourg@u-bordeaux.fr (K.D.); frederic.bauduer@u-bordeaux.fr (F.B.); 2 Laboratoire de Recherche en Management (LIREM), University of Pau and Pays de l’Adour, E2S UPPA, 64100 Bayonne, France; sandrine.cueille@univ-pau.fr; 3 Department of Physical and Rehabilitation Medicine, University of Toulouse 3, 31000 Toulouse, France; cf.roques@gmail.com

**Keywords:** spa therapy, balneotherapy, social network analysis (SNA), management

## Abstract

Spa therapy is a medical treatment based on the use of natural mineral water. In France, spa therapy is delivered in spa care facilities (SCF) involving the intervention of several actors (stakeholders). Spa doctors are key stakeholders as they prescribe the treatments, follow spa patients and assess spa therapy with scientific studies. This study aimed to analyze the spa doctors’ relationships in order to highlight their role in transferring information to other stakeholders, particularly to spa managers. For that purpose, we used the social network analysis (SNA) method by means of snowball data collection. We sent a questionnaire to all the spa therapy categories of actors. In total, 80 persons answered and declared 397 relationships. Our results, based on the categorization of respondents and of their relationships and on quantitative indicators (density, response rate), show that spa doctors demonstrate a very acceptable density of relations with spa managers and elected local authorities. However, they appear to be poorly involved in relations concerning the strategy and management of SCF, although they are essential actors in ensuring the medical relevance and sustainability of spa therapy. This research is of interest to patients’ care as it recommends deeper involvement of spa doctors in the management of SCF in order to optimize access to informational resources, specifically regarding the evolution of treatments in accordance with scientific progress. Our data are of international scope because the organizational model of balneotherapy, based on the cooperation between spa doctors and SCF, is universal.

## 1. Introduction

In France, spa therapy (or balneotherapy) is a medical treatment prescribed by doctors and reimbursed by Social Security. More than half a million patients are treated every year in 113 certified spa care facilities (SCF) located in 90 spa resorts for twelve medical “orientations” (musculoskeletal, respiratory, neurological, vascular, cardiac, digestive and metabolic, psychosomatic, urinary and metabolic, gynecological, cutaneous, pediatric, and oral health) [1].

The general practitioner or the specialist identifies the medical orientation and the location (name of the spa resort). The hydrotherapy protocol, determined and supervised by spa doctors, is delivered for 18 consecutive days over 3 weeks by certified caregivers. The number of treatments, their nature and technical characteristics are set for the twelve medical orientations by the national thermal convention (Journal Officiel République Française n° 0042—18 February 2023). SCF delivers care according to their particular agreements. Spa doctors carry out therapeutic techniques, especially for respiratory and dermatological disorders. They can also recommend additional support, for instance, patient education. Some of these doctors also take care of patients living in the area.

The reimbursement by Social Security is related to the demonstration of actual benefits to the patient for such treatments based on acceptable data of evidence. Several large randomized controlled trials have demonstrated the benefit of balneotherapy in several conditions: musculoskeletal conditions [2,3,4,5,6,7], venous conditions [8], stress-related disorders [9], overweight and metabolic syndrome [10,11,12], post-breast cancer [13,14,15] and neurodegenerative disorders [16]. Papers have also been published on “therapeutic patient education” for fibromyalgia [17], venous insufficiency [18], chronic low back pain [19], physical activity [20,21] or psychotropic withdrawal [22]. The medical literature also provides scientific evidence of resource-saving and less medication [9,23,24] and suggests fewer hospital stays [16,25] thanks to spa therapy.

Spa treatment is reimbursed at 65% and medical monitoring at 70% by Social Security, whatever the income conditions, representing approximately 0.18 to 0.20% of the Social Security national expenditure (*Objectif National de Dépenses d’Assurance Maladie*—ONDAM). Complementary health insurance funds cover the remainder. For about 75% of patients, the pocket expenditure averages EUR 1000 to 1200 as they live far from the resort [1,26]. Spa therapy activity represented, in 2019, a total of EUR 4.9 billion and 25,810 full-time equivalent jobs [26], generated either directly (EUR 500 million and 6600 jobs) or indirectly by suppliers (EUR 1.5 billion and 5670 jobs) and induced through the spending of people who work in SCF and their suppliers (EUR 2.9 billion and 13,540 jobs).

So, balneotherapy is a complex therapeutic intervention dedicated to patients mostly with chronic diseases that involve different categories of actors: patients, SCF administrators and technicians, spa doctors, technical and hygiene consultants, health authority officials, local authority officials, university and educational centers employees, hotel and campsites professionals, and technicians of water supply. Relations between these different categories of persons acting as key stakeholders have been poorly investigated [27] but are essential for the optimal management of the different material or intangible resources (related to competencies or those related to information [28]). The issue of the management of resources within firms or at the industry level has been largely investigated in the strategic management literature since the seminal work of Penrose (1959) [29] on the resource-based view (RBV) of the firm. Firms or industries that rely on the use of scarce resources with a low level of substitutability or resources regulated or controlled by powerful actors must develop management capacities to ensure their sustainable growth [30].

Social Network Analysis (SNA) is a method dedicated to the study of social networks, which are «a specific set of linkages among a defined set of persons, with the additional property that the characteristics of these linkages as a whole may be used to interpret the social behavior of the persons involved» [31]. However, a social network cannot be reduced to a sum of relationships. The social network is a “whole” of which the members are part. SNA is based on the concept of relational embeddedness [32], which makes it possible to take into account the effects of social structures on economic actions. Relational embeddedness means that the relationships between people and the structure of the network are an explanatory element of the economic fact. The action of individuals is then facilitated and limited, both by the structure and by the resources made available by the social networks [33]. SNA thus makes it possible to show the dependence of individuals with regard to their personal contacts. The SNA describes social structures to highlight the causes and consequences of the relationship networks [34,35], identifying the structures and their effects. This method has been used since the 1930s by sociologists and has then been of great interest to other sciences, such as physics, social sciences or management science, for instance [28]. By the 1990s, SNA had become an established field using specialized software, and social network research achieved a prominent position in management [36]. SNA can also be useful in medicine [37,38] but, to our knowledge, remains unused in spa therapy.

## 2. Materials and Methods

### 2.1. Spa Therapy in Les Landes

The use of SNA was made possible by the in-depth knowledge of the territory and the professionals involved in the activity of the Institute of Thermalism of Dax, which, since 2000, has been dedicated to the training of spa professionals (in 20 years: 1500 persons trained and 1250 graduated in the different diplomas) and to research.

**Figure 1:**
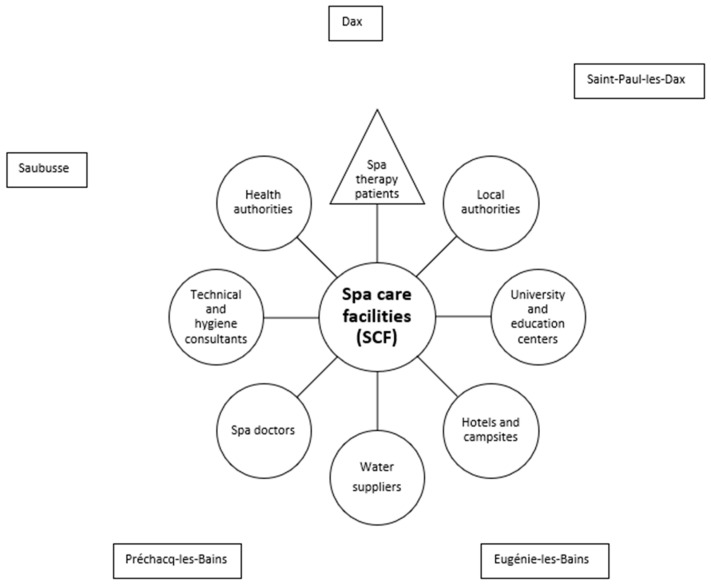
Stakeholders in the French spa therapy sector within *Les Landes*.

*Les Landes* represents a territorial entity from Southwestern France (so called “département” n°40 of 9243 km^2^ and 409,325 inhabitants in *Nouvelle-Aquitaine* administrative region) that demonstrates a high concentration in SCF: 18 SCF in 5 spa resorts (Dax, Saint-Paul-lès-Dax, Eugénie-les-Bains, Préchacq-les-Bains, Saubusse-les-Bains). *Les Landes* appears to be an exception in France, where there is generally only one SCF per spa resort, offering the possibility to question a particularly large number of professionals (stakeholders) (Figure 1). From a management point of view, the SCF managers are considered the central stakeholders.

### 2.2. Social Network Analysis Applied to Spa Therapy in Les Landes

SNA is based on sociometric data. Therefore, data collection consists of obtaining information on the relationships between individuals and the characteristics of these relations and the individuals. The choice of the method for collecting the social network depends on the approach of network boundaries definition [39]. Even if, in absolute, a network has no borders, in practice, the use of SNA is supposed to delimit the observed set [40]. The choice of the criterion applicable to establish the boundaries depends on the object of the research, which can be either the “complete network” or the “personal network”. When the boundaries of a social network exist *a priori*, inclusion rules are based on the common characteristics of actors or on common activities in which they participate (for example, membership in an organization). Thus, the so-called “complete network” approach consists in building the total social network of the population from the existing relationships between all individuals [41]. For this approach, three data collection methods, based on the point of view of an external observer, are possible: archives (documents showing the existence of relations between persons), artifacts (e-mails between persons, common participation at web social networks), and in situ observation. When the “complete network” approach is impossible, the social network is reconstituted by a snowball effect from the “personal network” (*Ego* network) of a first individual. This realistic way is based on the actors’ declarations of their perceived personal network. Indeed, a first individual quotes people with whom he or she is in relation, and then the people quoted are themselves questioned about their relationships, and so on. The decision to end this process determines the boundaries of the network studied. Then, two types of actors favor the closure of the social system: individuals having no relationship with the persons in the constituted network and individuals citing only actors already present in the constituted network. For the “personal network” approach, two data collection supports are possible: the questionnaire (“name generator”) and the self-survey [32].

SNA is then implemented on sociograms (graphical representations of the social networks) and on the calculation of network properties. Sociometric data are present in a table for the pairs of actors for whom a link has been recorded. However, they are most often entered in a square matrix, called an adjacency matrix, where individuals are listed in both rows and columns. The individuals in the rows are the starting vertices of relations, and the individuals in the columns are the arrival vertices of relations. The figure “1” indicates that the individuals are connected; “0” indicates that they are not. The matrix presentation is enriched by placing numerical values descriptive of the relationship (for example, the weight of the relationship) at the intersections of rows and columns. The sociogram created by Moreno [42] is made up of nodes and arrows connecting the nodes. In SNA, nodes are individuals characterized by a number of attributes (sex, age, function, etc.), and arrows are relationships qualified by their content, intensity and frequency [43]. In order to position the individuals of a network on the sociogram, sociometry uses many algorithms implemented by network representation software. They come from the graph theory that constitutes “the keystone” of the search for the properties of networks [44], providing SNA with a vocabulary to describe the structural properties of social relations. Moreover, it offers algorithms to allow the description, quantification and measure of relational properties [45].

The quantitative analysis of social networks completes the matrix and its graphic representation, thanks to the measurement of the characteristics of the network—nature of the relationship, structure of the social network and position of a person (Table 1).

In this study, we explore spa doctors’ relationships with other actors of balneotherapy by using the SNA. Our aim was to better analyze their roles in the management of SCF.

The first step was to choose the data collection method for the SNA. We used the “personal network” approach based on the individuals’ perception of their personal networks. As for the collection method, we chose the name generator (questionnaire). Based on our knowledge of the environment of the balneotherapy sector, we drafted a questionnaire divided into two parts. The first aims to collect personal and professional information about the respondents. The second includes the individuals with whom the respondent maintains essential professional relations for the sustainability and development of his activity. Some questions relate to the identification and professional activity of these individuals. Other questions characterize the relationships that the respondent has with these individuals. In order to avoid difficulties of understanding or inaccuracies that could slow down the respondent and lead to non-responses to the questionnaire, we submitted, to the criticism of a working group, the questions and possible answers. This working group, made up of seven professionals (two directors of SCF, one director of trade council, one elected local official, one public regional agent, one spa doctor, and one university agent), met for the first time in December 2014. Considering their comments, we modified our first version of the questionnaire. At this stage, we decided not to suggest a limited set of relationships to be declared so that each respondent could complete his list at his convenience, as suggested in the literature [46]. The members of the working group took part in a second meeting in January 2015 to comment on the survey website being created.

The purpose of the questionnaire thus finalized (Appendix A) was to identify the actors, the reasons for relations between them and the modalities of these relations (frequency, modes of relations, weight—scale from 1 to 10, duration). The questionnaire was self-administrated online, on a dedicated website, using a previously communicated password and guidelines. Its estimated duration was 30–40 min. The dedicated website mentioned the privacy of respondents: “Individual collected data are strictly confidential. They will be used for statistical purposes and only syntheses will be published. The processing of personal data collected on this website is the subject of a declaration to the CNIL (*Commission Nationale de l’Informatique et des Libertés*)”.

The data produced by the responses to the questionnaire were (i) the identification and description of the respondents by themselves (*Ego*) and (ii) their description of the people with whom they are connected (*Alter*) and of their relationship (*AlterRelation*). Thus, the weekly extraction of the database since the launch of the survey (29 June 2015) and the case-by-case processing of new relationships led to the distribution of the survey by snowball effect until the end of the diffusion of the questionnaire (30 October 2015).

In order to graphically represent the collected social network data and allow analysis, we have classified individuals and relationships using the R 3.2.1 for Windows statistical software (R Core Team, Vienna, Austria). Indeed, given the large number of variables characterizing individuals and relationships, we have classified *Egos* and *AlterRelations* in order to be able to distinguish them according to their classes. The statistical method of hierarchical classification [47] makes it possible to identify the classes of actors (subjects having similar characteristics) and the classes of relations (patterns of similar relations). The Cytoscape 3.4.0 software [48] materialized these class data in the graphical form of sociograms.

We used the social network density (proportion of relationships in a network, given the number of total possible relationships) as an indicator to describe the ability of actors to cooperate, a crucial issue for sharing information resources and providing cohesion [49]. The density (*δ*) is the ratio between the number of existing relationships (*L*) and the number of possible relationships. This last is the multiplication of the number of individuals (*g*) by the number of individuals minus 1 (*g* − 1), as it is impossible to be in relation with oneself. So, the density formula is:

(1) $$\delta =L÷\left(g\times \left(g-1\right)\right)$$

## 3. Results

In the first stage, 177 persons received the questionnaire. Some of the 177 individuals on the original list reported relationships with individuals on this list and relationships with 119 individuals not on this list. Among these 119 individuals, we selected 88 individuals who carried out their activity in the spa therapy sector or a related sector (hotels, prescribers, investors, etc.) in *Les Landes*. The 31 other persons were not selected as they did not practice in *Les Landes*. Overall, the invitation to respond to the questionnaire was sent to 265 persons. A total of 80 respondents reported 397 relations.

The classification of individuals and relationships using the R 3.2.1 for Windows software identified five *Ego* categories interpreted and plotted on a sociogram (Appendix B).

The response rate of spa doctors was the lowest, the highest being the elected local authority and the spa managers (Table 2).

We calculated the density for each category of *Ego*. The density of the spa doctors’ relations was higher than the density of spa managers and elected people (Table 3).

We retained four *AlterRelations* categories: (i) relations with providers facilitate the access to mineral water by SCF as this resource is essential and must be used locally and directly from the source; (ii) relations with public administration as they contribute to providing subventions; (iii) relations with spa doctors (key for all stakeholders in medical activity); (iv) relations for the exchange of information related to the strategic management of spa therapy activities (those do not concern relations with particular type of actors but rather define a particular type of information). We calculated for each *Ego* category the distribution of relations types (Table 4).

In Table 4, we can observe that spa doctors have mainly relations with spa doctors. Spa managers have equal *AlterRelations* with all the categories, and so do the elected people. Globally, three *Ego* categories declared the C *AlterRelation* category (relations with spa doctors): spa doctors, spa managers and elected people. This network analysis makes it possible to identify that approximately 90% of health issues are discussed by spa doctors, spa managers and elected people; however, spa doctors are poorly involved in D *AlterRelations* (exchange information related to the strategic management of spa therapy).

Among the categories of *Egos* that have the highest rates of relationships with spa doctors, spa managers and elected officials have a very high response rate (close to 90%). Spa doctors’ response rate (23%) is close to the global response rate (30%) (Table 2).

## 4. Discussion

### 4.1. The Social Network Analysis Technique for Studying the Spa Therapy Sector: Limits and Strengths

Our findings indicate that spa doctors demonstrate a very acceptable density of relations with spa managers and elected local authorities. However, they appear to be poorly involved in relations concerning the strategy and management of SCF. In order to discuss the role of spa doctors in the balneotherapy sector, we present the limits and strengths of SNA techniques implemented in balneology in *Les Landes*.

Spa therapy in *Les Landes* is studied in this research according to the qualitative case study methodology [50]. The construction of the questionnaire, the data collection and their analysis using SNA are valid for the case studied. They do not require statistical minimum data to ensure scientific quality. Therefore, we did not seek statistical generalization but rather an explanation of one situation. Since the population analyzed is limited, adding additional data may weaken the significance of the results. Subsequently, a study on a larger population would deepen and refine the results for a more focused objective. Moreover, we would be able to address and mitigate potential biases by incorporating quantitative measures such as Likert scales [51].

We could not use the “complete network” approach, as it was impossible to identify all categories of persons previously involved in the spa therapy sector. As for the collection method, we chose the questionnaire (name generator) rather than the self-survey, as the latter requires longer timing.

The survey by online questionnaire with the recruitment of respondents by a snowball effect seemed to be the most relevant method for collecting data from the social networks of spa therapy in *Les Landes*. However, even if the information technology (IT) solution facilitated our data collection, the success of this survey method depended on the willingness of the contacted people to participate and the ability to encourage them. We can explain the low participation rate by the reluctance to answer online questionnaires and the fact that some people who were contacted could not feel authorized to discuss their professional relations about the strategies of the organization.

We calculated the survey response rate using the system for monitoring invitations to participate. The response rate was 30% and may be considered rather low to draw valid conclusions. Indeed, from a theoretical point of view, the construction of a complete social network presupposes having obtained the participation of all members of the observed group [52]. However, a comparison with participation rates obtained in a few empirical cases of online surveys [53,54] allows us to consider that the rate we obtained is sufficient to implement an SNA. Depending on the population and the subject studied, the participation rates for online surveys are generally much lower than the rate obtained by other collection methods [55]. Thus, even if a participation rate of 40% is far from ideal, it can be considered satisfactory for recruiting respondents via e-mail [53]. The shares obtained are often lower, especially when respondents are unpaid; for example, the accepted participation in the survey of social networks of a competitiveness cluster conducted via the Internet was 23.5% [56].

The density of a social network is a prominent indicator, as dense networks facilitate the flow of information between individuals in the group [57]. The analysis of the density of a network is carried out simultaneously with the analysis of the “closure of a network” in order to understand their combined effects [58,59]. For a given group of individuals, the density of social relations and the “closure of the network” increase the sharing of resources between them. However, an open network can facilitate access to various resources [58]. In complement to the density of the network studied, it is relevant to analyze the diversity of areas of expertise of individuals in the network [49]. Analyzing the density and the closure of a social network together with the areas of expertise of individuals contributes to understanding the involvement of stakeholders in promoting or limiting access to information. In our study, spa doctors demonstrate a very acceptable density of relations with SCF managers and elected local authorities. Therefore, this is in favor of a satisfactory level of information-sharing between them, specifically regarding scientific progress and natural/medical resources issues. The diversity of expertise of these three key stakeholders (spa doctors, SCF managers and elected officials) and the openness of the network, thanks to their membership in national professional associations, give support to the dissemination of relevant information in order to access resources and medical information aiming at ensuring balneotherapy sustainability.

We used SNA in order to analyze stakeholders’ relationships in spa therapy, which is an original method in this field. To our knowledge, SNA was only used in one other research applied to balneology. This study investigated health policies in Turkey [60] and revealed that ensuring active relations between stakeholders in the network is critical for the development of the Turkish health sector.

Moreover, we note that innovative methods have also recently been applied to balneology research, such as Google Trends analysis [61] and YouTube video analysis [62].

### 4.2. The Role of Spa Doctors in the Balneotherapy Sector

This study provides a sample of relations in the French spa therapy sector: (i) in 2019, *Les Landes* hosted 75 401 spa therapy patients (total French spa therapy attendance: 579 630) [63], (ii) musculoskeletal orientation is the first medical orientation in *Les Landes* (as in France, 73% of attendance) [64], (iii) in *Les Landes*, all SCF are operated by private companies (85% in France). Alongside private family SCF, almost half of the balneotherapy activity in *Les Landes* is concentrated in the hands of groups (8 out of 18 SCF) as it is globally in France (53%) [26].

For the purpose of this study, we focus here on results concerning spa doctors among all stakeholders included in the balneotherapy sector [65].

Spa doctors were few to participate in the survey but these respondents showed a high density of relations, which means that their proportion of relationships in the network is high. Relations of spa doctors are half with their colleagues (40.5%) and the other half with spa managers (24.3%) and elected officials (24.3%). Hence, spa doctors mainly discuss with these three categories of stakeholders. Moreover, they have very few (2.7%) relations for the exchange of information related to the strategic management of spa therapy activities, which explains their poor involvement in the strategy and management of SCF. In consequence, the medical information transfers mainly between spa doctors and, to a lesser extent, from spa doctors to spa managers and elected officials. The relations for the exchange of information related to the strategic management of spa therapy activities are mainly a matter of elected officials (45.5%) and spa managers (30%).

Our SNA results are overall unequivocal. They identify the balneotherapy activity that is mainly based on three types of actors (spa doctors, spa managers and elected officials).

In France, spa doctors are prominent and play a central role in the field thanks to their expertise in medical hydrology [27], but this study highlights their insufficient involvement in the management of balneotherapy activity. The structure of the social networks unveiled by our study seems adequate concerning the relations that spa managers and elected people demonstrate with the other categories, thus facilitating the transfer of information between them. But, it may prove inadequate to allow SCF to gather informational resources concerning strategic management issues that, on top of material and competence-based resources, are necessary for their sustainable development. Spa doctors could favor the access to informational resources, specifically regarding the evolution of treatments in accordance with scientific progress, by developing their relations with spa managers. For ethical reasons, balneotherapy, as medical care, must be updated in accordance with scientific data. Thus, the current situation regarding the transfer from theory to practice is not optimal. Improving relationships between spa doctors and SCF managers could enhance the overall effectiveness of balneotherapy programs as spa doctors could communicate their observations and analyses regarding the evolution of patients, regulation, and natural and human resources. The public health benefits would be undeniable on two levels as the 18 days spent in SCF for spa therapy allow patients with chronic diseases to (i) lower pain and maintain functional capacity and quality of life [66,67,68] and (ii) adopt physical activities and eating behaviors useful to prevent comorbidities (cardiovascular disorders) [69]. Patient education is a therapeutic complement to the spa treatments that involve not only the spa doctors and the SCF but also the spa resort (elected representatives of the city, restaurants and shopkeepers). Unfortunately, due to the current medical demographic issue in France, clinical practice takes most of the spa doctors’ work time, and they cannot get involved in other activities. As the results presented in this paper should arouse spa doctors’ interest, they should be willing to participate in the continuation of this study.

## 5. Conclusions

We hope that the recruitment of salaried doctors, which has recently been made possible in France, will solve this problem. These doctors would be providers of essential intangible resources for SCF, and they would be more conveniently involved in the strategy definition and the management of the SCF.

In the future, this survey should be repeated to observe any changes. We could implement additional qualitative research, such as interviews or focus groups, in order to delve deeper into the reasons behind spa doctors’ limited engagement in strategic and managerial aspects of SCF. It could include nurses and physiotherapists whose presence is compulsory to run SCF.

Our results could give guidance to French balneotherapy, but this research is also relevant for balneotherapy worldwide as the organizational model based on the cooperation between spa doctors and SCF is universal.

Moreover, as balneotherapy uses natural resources (mineral and thermal waters, gases and mud) issued from the Earth [70] (and some are returned to the ground after elimination of all forms of danger and pollution), the preservation of the natural environment is of high interest for stakeholders in order to maintain the health value of spa therapy. Therefore, the ecological perspective will be addressed in our further research.

Finally, this work implemented in the field of spa therapy suggests the interest of SNA application to other health sectors.

## Figures and Tables

**Table 1:** Main indicators to measure social network characteristics.

Nature of the Relationship	Intensity: The Strength of the Relationship between Two Persons Reciprocity: The Degree of Common Perception of the Relation by the Two Persons
Structure of the network	Size: number of persons in the network Distance: number of relations between two persons Geodesic distance: minimal distance between two persons Diameter: longest geodesic distance Density: proportion of relationships in a network, given the number of total possible relationships Clique: group of persons related with reciprocal links Block: group of equivalent persons, i.e., they have the same kind of relations with other persons
Position of a person	Degree centrality: number of direct links between one person and the other persons in the network Betweenness centrality: ability of one person to link two other persons

**Table 2:** Response rate of *Ego* categories.

		Interpretation of *Ego* Categories	Invitations to Respond	Participants	Response Rate
*Ego* categories	1	Work in thalassotherapy	0	7	Non-applicable
	2	Spa managers	25	22	88%
	3	Spa doctors	47	11	23%
	4	Elected local authority	14	13	93%
	5	Work in public administration	30	16	53%
		Global participation	265	80	30%

**Table 3:** *Ego* classification and density of the spa therapy network in *Les Landes*.

Ego Categories	1	2	3	4	5	Total
Interpretation of Ego Categories	Work in Thalassotherapy	Spa Managers	Spa Doctors	Elected Local Authority People	Work in Public Administration	
Nb of *Egos*	7	22	11	13	16	69
Nb of *Alters*	26	91	40	97	95	349
Nb of *Alters*/*Ego*	3.71	4.13	3.63	7.46	5.93	5.06
Nb of Relations	23	110	34	118	112	397
Nb of *Relations*/*Ego*	3.29	5	3.09	9.08	7	5.92
Density	0.035	0.013	0.022	0.013	0.013	

**Table 4:** Relations between the actors of the spa therapy sector in *Les Landes*.

		*AlterRelation* Categories			
		A	B	C	D
	Interpretation of *AlterRelations* categories	Relations with providers	Relations with public administration	Relations with spa doctors	Relations for the exchange of information related to the strategic management of spa therapy activities
*Ego* categories	Work in thalassotherapy	7.8%	4.5%	2.7%	3.6%
	Spa managers	27.2%	27.3%	24.3%	30.0%
	Spa doctors	6.3%	6.8%	40.5%	2.7%
	Elected local authority	26.2%	11.4%	24.3%	45.5%
	Work in public administration	32.5%	50%	8.1%	18.2%
	**Total**	100%	100%	100%	100%

**Figure A1:**
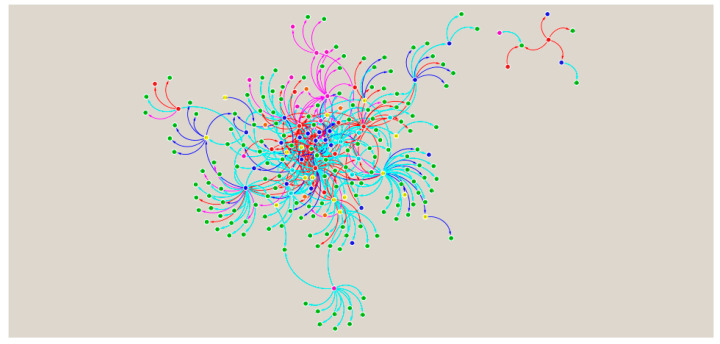
The sociogram of spa therapy in Les Landes.

## Data Availability

The datasets analyzed during the current study are available from the corresponding author upon reasonable request.

## Appendix A. The Questionnaire

**The Respondent**			
Personal information: first name, name, address, e-mail, phone number, age Professional information: duration of work experience in spa therapy, name of the organization in which I work, activity of this organization, kind of organization, my status in this organization, my duration in this organization, name of other organizations where I work			
**For each relationship**			
The person with whom I am in relation		The relation characteristics	
Identification:		For my professional occupation, the person I am in relation with is:	
	Sir or Madam First name Name E-mail Phone number		Competitor Partner Client Prescriber Supplier/subcontractor Control and regulatory organization Other
Professional information: Organization in which he/she works:			
Kind of organization:		Kind of exchanged information:	
	Small or medium enterprise Private group Self-employed (liberal activity) Professional unions Public company Local authority (region, department,...) State I do not know Other		Technical innovation Human resources Financing Administrative Marketing Development strategy (diversification, specialization, …) Clients Cooperation, joint-operation Regulatory framework Other
Activity sector of the organization in which he/she works:		Frequency of this relation:	
	Spa therapy Spa wellness Thalassothérapie Technical spa therapy Hotels, campsites, accommodation rentals,... Transport Retail, services Education and research Consulting Media Banking and finance Public administration Other		Several times per month Several times per year Once or twice per year Rarely
		Weight of this relation:	Indicate the relative importance of this relation compared to your others (from 1 to 10)

Location of this organization: town, postcode, country		Comments on this relation:	
Status of the person in this organization:			
	Managing director Manager Not manager Self-employed Elected (local, regional, national, European) Shareholder Other		
Name of the organization(s) in which he/she works:			
